# The Straight Position in Injuries of the Elbow-Joint

**Published:** 1893-11-11

**Authors:** A. Hanbury Frere


### The Hospital Clinic.

[*The Editor will be glad to receive offers of co-operation and contributions from members of the profession. All letters should be addressed to* The Editor, The Lodge, Porchester Square, London, W.]

### THE STRAIGHT POSITION IN INJURIES OF THE ELBOW-JOINT.

By A. Hanbury Frere, M.B.

The results of our present treatment of elbow fracture, in the rectangular position, are far from satisfactory. So long as they obtain good movement most surgeons seem to care nothing for deformity. But surely a time has now arrived when we ought to aim at something more than conserving the mobility of the joint only.

We find that attempts have been made from time to time to avoid some of the dangers attending the use of the rectangular position, showing that this method has not always given that satisfaction which would warrant its universal adoption. Thus Hippocrates and Sir A. Cooper were careful to keep the forearm prone. Swinburne* introduced a most ingenious method of extension and counter-extension; Bigelow† treated all injuries of the elbow, except fracture of the olecranon, "as though the forearm had been dislocated backward"; Watson Cheyne‡ has shown the propriety of operation. Amongst those who have abandoned the rectangular position, some favour the fully flexed position, but not only is this method open to the same objections as the rectangular position, but there is further a great interference with the circulation. Others prefer a position between extension and semi-flexion, but the success of this method does not appear to have been very great. Lastly, there are now many who believe that the best position in which the arm can be placed in these cases is in extension. So satisfactory has this method proved in the hands of those who have used it that I desire to consider briefly some of the advantages attaching to this plan of treatment, full details of which will be found in the works of Berthomier,§ Allis,‖ and Lane.¶

It is generally conceded that the cause of deformity or limitation of movement after elbow fracture is an alteration in the shape or relation of the articular surfaces, or an overgrowth of bone from excessive or ill-formed callus, or ossification of the ligaments or capsule.

Now all these changes are favoured by displacement of the fragments. What is wanted, therefore, is a method which will procure and keep up the best possible coäptation of the fragments, and at the same time preserve the normal angularity of the limb.

As Lauenstein** has pointed out, it is only in the extended position that we can be sure that the parts are in good position, and directly we bend the arm there may be great displacement or deformity, and we shall not be aware of the fact. What flexion conceals extension reveals. With the arm extended we can run the eye up the limb, and see that the proper relation of the parts is being kept up.

It is but natural, therefore, to retain the limb in that position in which we know that reduction is being kept up. There is no need to bend the arm, and thereby place the limb in such a position that we can only hope that all is well.

By extending the arm we practically eliminate muscular action, as we do away with the lever action that comes into play so soon as the limb is flexed. In the straight arm the structures on the flexor aspect of the joint are stretched to their physiological length. In this position, therefore, we obviate the danger of shortening, and there is less room for the formation of exuberant callus. At the same time, we are less likely to have adhesions or entanglement of the vessels and nerves, and these important structures will not be injured during performance of passive motion, as they are apt to be when healing has taken place in the flexed position.

In the extended position the ligaments become a valuable aid in steadying the fragments. I may here remark that the straight position is the best for fracture of the condyles of the femur, how much more is it suitable for fracture of the condyles of the humerus, where muscular action is not nearly so potent a factor? The extended position is most favourable to immobilisation, which is "the most efficient agent we possess against inflammation" (Stimson†), and which, at the same time, greatly aids the healing process.

With the arm extended there is no impediment to the circulation either of the blood or lymph, and it is only in this position that massage can be efficiently carried out.

Allis has shown that in the straight position we have much more control over the fragments than when the arm is bent.

In employing the extended position the forearm may be placed in supination or in pronation. Those who adopt the former claim that they are keeping up the normal outward obtuse angle at the elbow, but it is extremely painful to keep the arm supine for any length of time, and in such a position there is a danger of over-extension. Moreover, the angle at the elbow is formed by the humerus and ulna; it is still present, therefore, even when the forearm is prone.

Now, as Illingworth‡ has pointed out, with the arm extended and prone, the outward configuration of the limb becomes practically straight, so that, instead of a complicated angular splint, a simple straight one can be applied. Whilst, therefore, we are keeping up the normal relation of bone to bone, we have an arm that is much more easily dealt with than when the forearm is supine, and we avoid the danger of over-extension. With the arm extended and prone, the extensor muscles tend only to move the limb back as a whole, firm, rigid, and fixed as though it were composed of one long, straight bone.

It has been found that when the limb has been dressed in supination there is a tendency to pronation, which very soon loosens the dressing.

Another point in favour of pronation is the fact that the limb is all ready to be bent to a right angle without any rotation of the humerus; there will thus be less strain on the freshly united structures when the arm is moved for the first time, should we then desire to place the arm in a sling.

Lastly, as Morris§ says, "pronation is a position of the greatest elegance and grace, and one most agreeable to the eye as well as to the feelings."

Any tendency to swelling of the hand may be avoided by careful bandaging, and such a position is not more uncomfortable than any other in such an injury.

It is a great point to dress the limb in that position in which the fracture is best set, for in this way we greatly hasten the healing process, and thus dressings can sooner be dispensed with.

I believe that an exaggerated fear of anchylosis, by perpetuating the use of the rectangular position, has been the chief impediment to the adoption of the straight position. If one position more than another is favourable to anchylosis it is the rectangular position. If the injury be so severe as to render anchylosis

* Med. and Surg. Reporter. Philadelphia. October 26th, 1861.

† Boston Med. and Surg. Journ. May 7th, 1868.

‡ B.M.J. March 7th, 1891.

§ Mechanism des Fractures du coude chez les enfants. Leur traitement par l'extension. Paris. 1875.

‖ Annals Anat. and Surg. Society. Brooklyn. II. No. 8, 1880.

¶ Transactions American Surg. Assoc. IX. 1891.

** See précis by Mr. Nunn, *Lancet*, March, 1889.

† Transactions American Surg. Assoc. LX. 1891.
‡ Brit. Med. Assoc. Dublin, 1887. § Anatomy of the joints of man.

almost inevitable, an attempt should be made to save the joint by operation. But such cases are not of sufficient frequency to make this an argument in favour of the universal use of the bent position. We know that fracture of the elbow is often overlooked, and fracture of the olecranon has been put up at a right angle with disastrous results. It is, therefore, as a routine practice that the straight position especially commends itself. It has advantages possessed by no other method, and were the extended position to become the rule instead of the exception, it would be the means of preventing much pain to the patient, and the saving of much time, trouble, and anxiety to the general practitioner.

